# Prevalence of Fast Food Intake among a Multi-Ethnic Population of Young Men and Its Connection with Sociodemographic Determinants and Obesity

**DOI:** 10.3390/ijerph192214933

**Published:** 2022-11-13

**Authors:** Jozaa Z. AlTamimi, Naseem M. Alshwaiyat, Hana Alkhalidy, Nora A. AlFaris, Nora M. AlKehayez, Malak A. Alsemari, Reham I. Alagal

**Affiliations:** 1 Department of Physical Sports Sciences, College of Education, Princess Nourah bint Abdulrahman University, P.O. Box 84428, Riyadh 11671, Saudi Arabia; 2 School of Nutrition and Dietetics, Faculty of Health Sciences, Gong Badak Campus, Universiti Sultan Zainal Abidin, Kuala Nerus 21300, Terengganu, Malaysia; 3 Department of Nutrition and Food Technology, Faculty of Agriculture, Jordan University of Science and Technology, Irbid 22110, Jordan; 4 Department of Medical Imaging—MRI, King Abdullah bin Abdulaziz University Hospital (KAAUH), Princess Nourah bint Abdulrahman University, P.O. Box 84428, Riyadh 11671, Saudi Arabia; 5 Department of Health Sciences, College of Health and Rehabilitation Sciences, Princess Nourah bint Abdulrahman University, P.O. Box 84428, Riyadh 11671, Saudi Arabia

**Keywords:** fast food, multi-ethnic, young men, sociodemographic, obesity

## Abstract

Fast food is commonly consumed by young adults. Eating fast food is connected with the risk of obesity and other related diseases. The present study examines the prevalence of fast food intake in a diverse sample of young men. This cross-sectional study included 3600 young men (20–35 years) who resided in Riyadh, KSA. The frequency of fast food intake was assessed using a valid and reliable questionnaire. Weekly and daily intake of fast food were the two outcome variables adopted to assess the intake frequency. Weight and height were measured. Fast food was eaten by 88.8% and 50.1% of participants weekly and daily, respectively. Fast food intake was predicted by the nationality of participants. The highest prevalence of weekly fast food intake (99.7%) was observed among Saudi, Egyptian, and Indian participants, while the lowest rate was observed among Sudanese participants (48.6%). The highest and lowest rates of daily intake were seen among Filipino (83.4%) and Bangladeshi (6.3%) participants. Obesity was another predictor of fast food intake. Obese participants had a significantly higher odds ratio of weekly (OR = 2.89, *p* = 0.006) and daily (OR = 1.39, *p* = 0.021) fast food intake than non-overweight/non-obese participants. In conclusion, fast food is frequently consumed by young men in KSA. Our findings link the likelihood of fast food intake to sociodemographic determinants and obesity.

## 1. Introduction

Fast food intake has rabidly exploded at the global level, especially among young adults [1]. Fast food is generally known as ultra-processed food with low nutritional value that can be easily accessible from restaurants. Commonly eaten fast food includes burgers, French fries, fried chicken, and pizza [2]. Fast food is usually prepared by adding excessive fat, saturated fat, refined carbohydrates, and sodium. Therefore, eating fast food increases ingested calories and lowers overall diet quality [3,4]. Fast food intake is linked to a high incidence of various illnesses, including obesity, diabetes, stroke, and colorectal cancer [5,6,7,8,9]. Fast food is typically nutrient-poor and energy-dense food, therefore eating it frequently could result in weight gain and higher obesity risk [5]. Furthermore, a higher incidence of type 2 diabetes mellitus and a higher mortality rate from cardiovascular disease and stroke are both related to an increase in fast-food restaurant density [6,7]. Weight gain associated with fast food consumption may cause an increase in body adiposity. Increased adiposity is a significant risk factor for metabolic abnormalities, such as insulin resistance and serum lipids disturbances, which accelerate the development of type 2 diabetes mellitus and cardiovascular disease [7]. Fast food intake is linked to increased weight and waist circumference, higher insulin resistance scores, higher plasma triglycerides levels, and lower high-density lipoprotein cholesterol levels [8]. Eating fast food is associated with higher consumption of saturated fatty acids, trans-fatty acids, cholesterol, refined carbohydrates, and sodium, and lower consumption of dietary fiber, micronutrients, and antioxidants such as ascorbic acid and carotenes. Therefore, poor diet quality could be another mechanism to connect fast food intake and the risk of noncommunicable diseases [7]. In addition, eating fast food is linked to a higher risk of colorectal cancer incidence due to the buildup of carcinogenic compounds, such as polycyclic aromatic hydrocarbons and dietary trans-fatty acids, yields from the method of fast food preparation and cooking [9]. Currently, fast food consumption is viewed as a critical public health concern [10]. The WHO was prompted by this circumstance to develop recommendations for individuals to keep their daily calorie consumption of fat and saturated fat to less than 30% and 10%, respectively [11]. 

Furthermore, several studies have reported a relationship between fast food intake and sociodemographic determinants, including age [12,13], ethnicity or nationality [14,15], marital status [16], educational level [14,17], and family income [16,18]. Although the literature provides consistent results about the relationship between some sociodemographic determinants and fast food intake, it provides contradictory findings about the connection between other sociodemographic determinants and fast food consumption. For example, aging has been linked with a lower frequency of fast food intake in the literature [12,13]. On the other hand, fast food consumption has been connected with lower educational levels and lower family income in several reports [16,17], while opposite relationships appear elsewhere [14,18]. Therefore, it is important to investigate the relationship between different sociodemographic determinants and fast food intake for different populations to gain a deep understanding of the role of sociodemographic determinants in motivating adults to eat fast food. In any case, adults frequently consume fast food; thus, it is important to look into trends in fast food consumption and associated sociodemographic determinants to develop effective health promotion plans to reduce their intake at the community level, with a focus on high-intake population subgroups [19].

Obesity has become a health crisis in the Kingdom of Saudi Arabia (KSA), which is also home to poor lifestyle habits including sedentary behaviors and a diet high in fast food [2,20]. Many international brands, such as McDonald’s, KFC, and Pizza Hut, and many national brands, such as Kudu, Albaik, and Maestro Pizza, are chains of fast-food restaurants operating in KSA. In fact, KSA is the region’s largest oil producer, and its economy is expanding swiftly. Consequently, KSA employs people from all over the world. In reality, nearly 50% of the manpower in KSA and nearly 90% of the jobs in private businesses are performed by foreigners [21]. Foreigners constitute about 30% of the population in KSA, with about three-fourths of them being male [22]. Exploring the differences in eating behaviors and their connection to health in a diverse population is made intriguing by the presence of immigrants of different ethnic origins. In addition, men consumed fast food more often than women [19]. Hence, the purpose of our study was to assess the prevalence of weekly and daily fast food intake in a diverse sample of young men and the connection between fast food intake and sociodemographic determinants and obesity.

## 2. Materials and Methods

### 2.1. Participants and Study Design

Data for the present study were extracted from a research project called Relationship between Obesity, physical Activity and Dietary pattern among men living in the Kingdom of Saudi Arabia (ROAD-KSA) [23,24,25,26,27,28,29]. This research project was designed to determine the prevalence of overweight and obesity, physical activity levels, and dietary patterns among young and middle-aged men from twelve Middle Eastern and Asian countries living in Riyadh, Saudi Arabia. This cross-sectional study was conducted in Riyadh, KSA. Following a stratified clustered selection method, participants were randomly selected from open venues in Riyadh. The eligibility criteria included young men (20–35 years) residing in the city, devoid of any disability, and being citizens of one of the twelve countries (see Table 1). Written consent was acquired from subjects following the principles of the Helsinki Declaration. Princess Nourah bint Abdulrahman University’s research ethics committee approved the current work.

### 2.2. Sociodemographic Determinants

Skilled researchers filled in the sociodemographic data from recruited subjects. Data were collected following one-to-one interview method.

### 2.3. Overweight and Obesity Determination

Skilled researchers measured the weight and height of subjects with light clothes, barefoot, and standing fully upright. Weight was taken to the closest 100 g using a weight balance. Height was taken to the closest 1 mm using a stadiometer. Body mass index (BMI) values were determined from the weight and height [BMI = weight (kg)/height (m^2^)]. Subsequently, a BMI value equal to 25–29.9 was considered overweight, and a BMI value equal to or exceeding 30 was considered obese [30].

### 2.4. Instrument

A valid and reliable questionnaire was utilized to assess the fast food intake frequency of participants. The questionnaire was adopted from a previous study [12] and modified to suit the culture in KSA. The face validity of the instrument was independently assessed by five health research professionals. To evaluate the instrument reliability, a pilot study was conducted. The target population was used to recruit 60 volunteers, who were not included later in the study sample, for the test–retest pilot study with two weeks suspension. The Spearman’s correlation between the instrument test and retest results for fast food intake frequency was satisfactory (r = 0.86, *p* ˂ 0.05).

### 2.5. Fast Food Intake Assessment

Data were gathered by skilled researchers using one-to-one interview method. In the present study, fast food was determined as low nutritional value food that is easily accessible from restaurants. Therefore, fast food was grouped into eight types: burgers, French fries, pizza, fried chicken, hotdogs, pastries, shawarma, and falafel. One serving of each type was determined based on the average amount served in a single regular meal by restaurants. Thus, the serving size was determined as one regular burger (5 oz), one medium serving of French fries (4 oz), one small pizza (5 oz), one piece of fried chicken (6 oz), one hotdog sandwich (5 oz), one pastry (4 oz), one shawarma sandwich (5 oz), and one falafel sandwich (6 oz). The subjects were asked about the number of servings from each fast food type typically consumed per week or day over a year. Weekly and daily intakes were determined based on the total number of consumed fast food servings. Eating one serving or more per week was specified as a weekly intake while eating one serving or more per day was specified as a daily intake.

### 2.6. Statistical Analysis

SPSS software (version 26) was used for data analysis. Weekly and daily intake of fast food were the two dichotomous outcome variables adopted in the present study. A chi-square test was run to analyze categorical variables, and the result was displayed as frequencies (N) and percentages (%). A one-way ANOVA test was run to analyze continuous factors, and the result was shown as the mean (SD). After adjustment for sociodemographic variables and weight status, multivariate regression analysis was carried out to explore factors related to the intake of fast food. *p* values were extracted from a two-tailed test with a significance level of *p* ˂ 0.05.

## 3. Results

In sum, 3600 young men who reside in KSA and are from different countries were recruited for this study. Table 1 displays the prevalence of weekly and daily fast food eating among participants, broken down by sociodemographic determinants and weight status. Weekly fast food intake was indicated by 88.8% of participants, while daily fast food intake was indicated by 50.1% of them. Significant differences (*p* ˂ 0.001) in weekly and daily fast food intake rates were seen after subjects were separated into groups based on nationality. The participants from KSA, Egypt, and India had the greatest prevalence of weekly fast food intake (99.7%), while the subjects from Sudan had the least prevalence (48.6%). Moreover, the greatest prevalence of daily fast food intake (83.4%) was reported among Filipino subjects, while the least prevalence (6.3%) was reported among Bangladeshi subjects. The rate of weekly fast food consumption was significantly greater among subjects who had resided in KSA for at minimum six years (92.2%) compared to participants who had a lower residency period (86.6%). Residing with family was associated with a significantly higher prevalence of weekly (98.7%) and daily (73.1%) fast food intake than residing within non-family households (86.5% and 44.8%, respectively). Single participants had significantly higher weekly (91.1%) and daily (52.3%) intake rates of fast food intake than married participants (86.1%, and 47.7%, respectively). Remarkably, a high education level was associated with significantly greater weekly (98.5%) and daily (73.9%) fast food intake rates than a low education level (83.2%, and 36.4%, respectively). Similarly, a high monthly income was associated with significantly greater weekly (98.7%) and daily (63.6%) consumption rates of fast food than a low monthly income (85.1%, and 45.1%, respectively). Overweight subjects had significantly higher rates of weekly (90.6%) and daily (51.7%) fast food consumption than non-overweight/non-obese subjects (86.5%, and 48.1%, respectively). Moreover, obese subjects had significantly higher rates of weekly (95.9%) and daily (56.3%) fast food consumption than non-overweight/non-obese subjects (86.5%, and 48.1%, respectively).

**Figure 1:**
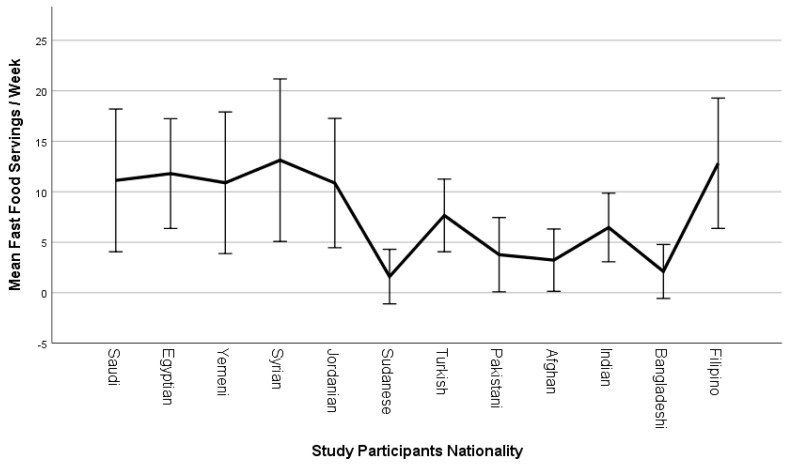
Line chart illustrating the mean number of fast food servings consumed per week among study participants stratified based on their nationality (mean ± SD).

The mean number of fast food servings consumed weekly differed significantly (*p* ˂ 0.001) between participants stratified based on their nationality (Figure 1). The mean number of weekly fast food servings was relatively high among participants from Syria (13.1 ± 8.0), the Philippines (12.8 ± 6.5), Egypt (11.8 ± 5.4), KSA (11.1 ± 7.1), Yemen (10.9 ± 7.0), and Jordan (10.9 ± 6.4), moderate among participants from Turkey (7.7 ± 3.6) and India (6.5 ± 3.4), and low among participants from Pakistan (3.8 ± 3.7), Afghanistan (3.2 ± 3.1), Bangladesh (2.1 ± 2.7), and Sudan (1.6 ± 2.7).

The odds ratios for participants’ weekly and daily fast food intake for sociodemographic determinants and weight status are demonstrated in Table 2. It was revealed that the nationality of participants could predict weekly and daily fast food intake. Compared to Sudanese participants, those from other countries had significantly higher likelihoods of weekly fast food intake (odds ratio [OR] = 3.60–496.43, *p* ˂ 0.001). In comparison with Bangladeshi participants, participants from other countries (except Sudan) had significantly higher likelihoods of daily fast food intake (OR = 2.20–58.84, *p* ˂ 0.01). In addition, more advanced age was significantly associated with a lower likelihood of daily fast food intake (OR = 0.96, *p* = 0.006). Subjects who had lived in KSA for six years or more had a significantly higher likelihood of weekly fast food intake than subjects who had lived there for five years or less (OR = 1.77, *p* ˂ 0.001). Participants who resided in family households had significantly higher likelihoods of weekly (OR = 2.38, *p* = 0.038) and daily (OR = 1.55, *p* = 0.002) fast food intake than participants who resided in non-family households. Married participants had a significantly lower likelihood of eating fast food per week than single participants (OR = 0.50, *p* ˂ 0.001). More highly educated participants had a significantly higher likelihood of fast food intake per day than lees educated participants (OR = 1.34, *p* = 0.020). Participants who earned higher incomes had a significantly higher likelihoods of weekly (OR = 3.20, *p* = 0.002) and daily (OR = 2.13, *p* ˂ 0.001) fast food intake than participants with lower monthly incomes. Lastly, overweight subjects had a significantly higher likelihood of weekly (OR = 1.85, *p* ˂ 0.001) and daily (OR = 1.16, *p* = 0.035) fast food intake than non-overweight/non-obese participants, while obese subjects had a significantly greater likelihood of weekly (OR = 2.89, *p* = 0.006) and daily (OR = 1.39, *p* = 0.021) fast food intake than non-overweight/non-obese subjects.

## 4. Discussion

The present study investigated the prevalence of fast food intake in a diverse sample of young men. Findings indicated that fast food intake was relatively high per week (88.8%) and per day (50.1%). The rates of fast food intake among adults have been reported in previous studies. A recent study found that 85.2% of young Saudi adults consumed fast food once per week at minimum [31]. Analysis of Nutrition Examination Survey 2017−2018 data revealed that fast food was eaten by 48.6% of young American adults daily [13]. Another study indicated that 60.4% of young men from Jordan eat fast food twice weekly at minimum [32]. A study from India found that 85% of young adults were fast food consumers, and 17% of them were daily consumers [33]. The prevalence of weekly fast food intake was 55.9 % among young men from Bangladesh [34]. Another study reported that 41% of Malaysian adults (24–49 years) were weekly fast food consumers [14].

The results of this study demonstrated that the prevalence of fast food intake varied based on the subjects’ nationality. Our finding is consistent with past studies that discovered a significant difference in fast food intake among individuals of different nationalities or ethnicities [13,14,15]. A recent report studied fast food intake frequency among adults from six countries and observed that the rate of fast food intake three times weekly or more among adults from Argentina (16.3%) was higher than that seen among adults from Croatia (13.0%), Romania (7.4%), Portugal (5.2%), Latvia (3.9%), and Hungary (3.7%) [15]. American research reported that the rate of adults’ fast food intake on a typical day for Blacks (42.6%) was greater than that for Whites (36.5%), Mexican Americans (34.9%), and Asians (33.8%) [13]. A report from Malaysia observed that the rate of weekly fast food intake among Malay adults (86.8%) was significantly higher than that observed among Chinese adults (9.6%) and Indian adults (3.6%) [14]. Variations in fast food intake rates by nationality could be referred to as differences in environmental exposure, such as fast food availability and accessibility, either in KSA or in their home countries [35,36]. Furthermore, eating fast food could be mediated by cultural standards and social values of adults based on their ethnic background [37]. 

This study revealed that the likelihood of daily fast food intake decreases with aging, and this result is in harmony with many earlier studies [12,13,14,16,17,18]. In agreement with the literature, the likelihood of fast food intake is increased with being single [16]. In KSA, married men frequently consumed home-cooked food, which is usually prepared by wives or female housemaids, while most single men found fast food a tasty and convenient meal compared with home food. Some striking results could be seen in this study. For example, living with family, having a higher education level, and having a higher income are associated with higher fast food intake. This is in stark contrast to the major findings found in the literature of developed countries, which show that people with a lower education levels and lower income would have higher fast food consumption [17,38,39,40]. However, the available literature provides controversial findings regarding the relationship between fast food intake and these determinants. Several studies have reported no association between fast food intake and education level [12,41,42,43], while other studies found that fast food intake was associated with higher education levels [14,44]. Likewise, a study found that fast food intake was associated with higher income [18], while another study found that fast food intake was not associated with living with a family [32]. In KSA, young men usually look to fast food as an important feature of the westernized lifestyle which is considered by them superior to the traditional lifestyle. Thus, fast food symbolizes high social status, so it attracts people with higher income and education levels to consume fast food. Furthermore, most families with higher income and education levels depend on foreign housemaids to cook and prepare food which is mostly not enjoyed by young people. Thus, they usually prefer to eat fast food as an appetizing alternative to home-cooked food. In addition, many educated young men in KSA have long work hours during the day, which makes them frequently consume fast food due to its high availability and accessibility, especially with the ubiquitous delivery services. 

Obesity is associated with a high risk of many diseases and health disorders among adults [45,46]. Remarkably, our results confirmed the association between fast food intake and obesity. The link between fast food intake and obesity is supported by numerous previous studies [5]. During the past few decades, fast food has grown in popularity, and its availability has increased greatly. Adults who are more exposed to fast food tend to eat fast food more frequently, which is called an obesogenic environment [47,48]. This results in an apparent increase in daily caloric intake from fast food, especially when served in large portions [49]. Young adults consume, on average, 19% of their daily caloric intake or about 425 kcal per day from fast food [13]. In addition, fast food intake among adults is mainly mediated by taste, satisfaction, and convenience [50]. Obesity is usually connected with dietary patterns characterized by food cravings [51]. Increasing BMI is positively associated with craving high-fat foods like fast food [52,53]. Obese people experience impaired control of appetite and the reward system caused by endocrine disturbances such as insulin resistance and decreased leptin release. The development of obesity has been linked to reward deficiency syndrome, which is characterized by a dysfunction in the dopamine D2 receptor, which mediates the rewarding feature of tasty foods [54]. Overall, fast food intake affects obesity development reciprocally.

Eating at fast-food restaurants has transformed into a type of social behavior and a contemporary lifestyle [50]. The public health sector has a major obligation to control the spread of this problem. Increasing community awareness of the detrimental health influences of eating fast food through tailored education programs may support attempts to limit the prevalence of fast food consumption [55]. In contrast, nutritious foods that contain fruits, vegetables, and whole grains should be promoted as a good replacement for fast food [56]. Limiting the availability of fast-food restaurants in surrounding areas of schools, universities, public gardens, and shopping centers may reduce exposure to fast food and lower its intake [57]. Moreover, a ban can be applied to home delivery services and fast-food advertisements [58,59]. Finally, forcing fast-food restaurants to add nutritional facts to food menus could help consumers to be aware of the caloric contents of fast food [60].

Some limitations could be found in the present study. The cross-sectional design prevented the utilization of significant associations to infer causality. Intake data were gathered using the frequency questionnaire method, which could underestimate fast food intake frequency as it depends on participants’ memory. Comparing our findings with those of earlier studies is challenging because fast food is defined differently among different studies. Finally, we recognize the lack of caloric intake data, which is not considered when analyzing the connection between fast food intake and obesity. However, this study provides insightful information on the rates of fast food intake and associated determinants.

## 5. Conclusions

The prevalence of fast food intake among young men who reside in Saudi Arabia was relatively high. The results prove a connection between the likelihood of fast food intake and sociodemographic determinants, including nationality, age, residency period in the country, household type, marital status, educational level, and monthly income, as well as overweight and obesity. Our findings revealed that examining sociodemographic determinants is key to planning effective preventative strategies. This is one of few studies investigating the prevalence of fast food intake among a multi-ethnic population of participants, while previous studies have mainly focused on those with a specific ethnic background. Therefore, recruiting participants with multi-ethnic backgrounds should be considered in future studies.

## Figures and Tables

**Table 1:** Prevalence of weekly and daily fast food intake by study participants (n = 3600) stratified by sociodemographic determinants and weight status.

Variables	Total N	Weekly Intake		Daily Intake	
		Yes N (%)	No N (%)	Yes N (%)	No N (%)
All Participants	3600	3196 (88.8%)	404 (11.2%)	1804 (50.1%)	1796 (49.9%)
Nationality					
Saudi	289	288 (99.7%)	1 (0.3%)	202 (69.9%)	87 (30.1%)
Egyptian	289	288 (99.7%)	1 (0.3%)	238 (82.4%)	51 (17.6%)
Yemeni	335	328 (97.9%)	7 (2.1%)	232 (69.3%)	103 (30.7%)
Syrian	293	290 (99.0%)	3 (1.0%)	229 (78.2%)	64 (21.8%)
Jordanian	280	279 (99.6%)	1 (0.4%)	201 (71.8%)	79 (28.2%)
Sudanese	276	134 (48.6%)	142 (51.4%)	24 (8.7%)	252 (91.3%)
Turkish	203	202 (99.5%)	1 (0.5%)	113 (55.7%)	90 (44.3%)
Pakistani	306	243 (79.4%)	63 (20.6%)	68 (22.2%)	238 (77.8%)
Afghan	303	247 (81.5%)	56 (18.5%)	40 (13.2%)	263 (86.8%)
Indian	297	296 (99.7%)	1 (0.3%)	119 (40.1%)	178 (59.9%)
Bangladeshi	350	226 (64.6%)	124 (35.4%)	22 (6.3%)	328 (93.7%)
Filipino	379	375 (98.9%)	4 (1.1%)	316 (83.4%)	63 (16.6%)
*p* value *		**˂0.001**		**˂0.001**	
Residency Period in KSA					
1–5 years	2198	1904 (86.6%)	294 (13.4%)	1100 (50.0%)	1098 (50.0%)
6 years or more	1402	1292 (92.2%)	110 (7.8%)	704 (50.2%)	698 (49.8%)
*p* value *		**˂0.001**		**0.921**	
Household Type					
Non-family household	2920	2525 (86.5%)	395 (13.5%)	1307 (44.8%)	1613 (55.2%)
Family household	680	671 (98.7%)	9 (1.3%)	497 (73.1%)	183 (26.9%)
*p* value *		**˂0.001**		**˂0.001**	
Marital Status					
Single	1919	1749 (91.1%)	170 (8.9%)	1003 (52.3%)	916 (47.7%)
Married	1681	1447 (86.1%)	234 (13.9%)	801 (47.7%)	880 (52.3%)
*p* value *		**˂0.001**		**0.006**	
Education Level					
Low (High school or less)	2284	1900 (83.2%)	384 (16.8%)	831 (36.4%)	1453 (63.6%)
High (College or more)	1316	1296 (98.5%)	20 (1.5%)	973 (73.9%)	343 (26.1%)
*p* value *		**˂0.001**		**˂0.001**	
Monthly Income					
Low (˂1000 USD)	2630	2239 (85.1%)	391 (14.9%)	1187 (45.1%)	1443 (54.9%)
High (≥1000 USD)	970	957 (98.7%)	13 (1.3%)	617 (63.6%)	353 (36.4%)
*p* value *		**˂0.001**		**˂0.001**	
Weight Status					
Non-overweight/non-obesity	1860	1608 (86.5%)	252 (13.5%)	894 (48.1%)	966 (51.9%)
Overweight	1518	1375 (90.6%)	143 (9.4%)	785 (51.7%)	733 (48.3%)
Obesity	222	213 (95.9%)	9 (4.1%)	125 (56.3%)	97 (43.7%)
*p* value *		**˂0.001**		**0.018**	

* Categorical variables were analyzed by using the chi-squared test and expressed as numbers and percentages. Significant values (*p*-value ˂ 0.05) are presented in **bold type.**

**Table 2:** Odds ratios of weekly and daily fast food intake by study participants according to sociodemographic determinants and weight status.

Variables	Weekly Fast Food Intake		Daily Fast Food Intake	
	Odds Ratio (95% CI) *	*p* Value	Odds Ratio (95% CI) *	*p* Value
Nationality				
Saudi	53.95 (7.03–414.39)	**˂0.001**	24.05 (13.38–43.24)	**˂0.001**
Egyptian	194.70 (26.49–1431.09)	**˂0.001**	58.84 (33.74–102.61)	**˂0.001**
Yemeni	39.64 (17.63–89.17)	**˂0.001**	30.30 (18.29–50.18)	**˂0.001**
Syrian	48.06 (14.43–160.01)	**˂0.001**	41.61 (23.94–72.34)	**˂0.001**
Jordanian	183.88 (24.86–1360.19)	**˂0.001**	34.23 (19.76–59.28)	**˂0.001**
Sudanese	1.00		1.37 (0.75–2.52)	**0.309**
Turkish	231.31 (31.55–1695.84)	**˂0.001**	22.11 (13.06–37.43)	**˂0.001**
Pakistani	4.57 (3.04–6.88)	**˂0.001**	4.01 (2.40–6.72)	**˂0.001**
Afghan	5.56 (3.70–8.35)	**˂0.001**	2.20 (1.27–3.82)	**0.005**
Indian	496.43 (68.12–3617.88)	**˂0.001**	9.58 (5.85–15.69)	**˂0.001**
Bangladeshi	3.60 (2.46–5.25)	**˂0.001**	1.00	
Filipino	103.05 (34.34–309.23)	**˂0.001**	56.72 (32.97–97.59)	**˂0.001**
Age (years)	1.01 (0.97–1.07)	0.581	0.96 (0.93–0.99)	**0.006**
Residency Period in KSA				
1–5 years	1.00		1.00	
6 years or more	1.77 (1.31–2.39)	**˂0.001**	1.08 (0.88–1.33)	0.443
Household Type				
Non-family household	1.00		1.00	
Family household	2.38 (1.05–5.42)	**0.038**	1.55 (1.17–2.04)	**0.002**
Marital Status				
Single	1.00		1.00	
Married	0.50 (0.37–0.66)	**˂0.001**	0.93 (0.76–1.14)	0.506
Education Level				
Low (High school or less)	1.00		1.00	
High (College or more)	1.86 (0.94–3.67)	0.076	1.34 (1.05–1.72)	**0.020**
Monthly Income				
Low (˂1000 USD)	1.00		1.00	
High (≥1000 USD)	3.20 (1.51–6.78)	**0.002**	2.13 (1.83–2.47)	**˂0.001**
Weight Status				
Non-overweight/non-obesity	1.00		1.00	
Overweight	1.85 (1.38–2.47)	**˂0.001**	1.16 (1.01–1.33)	**0.035**
Obesity	2.89 (1.35–6.16)	**0.006**	1.39 (1.05–1.84)	**0.021**

* Multivariate logistic regression analysis was used after adjusting for subjects’ sociodemographic variables and obesity. Differences were considered statistically significant at *p*-value < 0.05, and significant values are presented in **bold type**.

## Data Availability

Data are available from the corresponding author on reasonable request.
